# *In Vivo* Determination of Direct Targets of the Nonsense-Mediated Decay Pathway in *Drosophila*

**DOI:** 10.1534/g3.113.009357

**Published:** 2014-01-15

**Authors:** Alex Chapin, Hao Hu, Shawn G. Rynearson, Julie Hollien, Mark Yandell, Mark M. Metzstein

**Affiliations:** * Department of Human Genetics, University of Utah, Salt Lake City, Utah 84112; † Department of Biology, University of Utah, Salt Lake City, Utah 84112

**Keywords:** Upf2, reactivation, NMD, *Drosophila*, RNA-seq

## Abstract

Nonsense-mediated messenger RNA (mRNA) decay (NMD) is a mRNA degradation pathway that regulates a significant portion of the transcriptome. The expression levels of numerous genes are known to be altered in NMD mutants, but it is not known which of these transcripts is a direct pathway target. Here, we present the first genome-wide analysis of direct NMD targeting in an intact animal. By using rapid reactivation of the NMD pathway in a *Drosophila melanogaster* NMD mutant and globally monitoring of changes in mRNA expression levels, we can distinguish between primary and secondary effects of NMD on gene expression. Using this procedure, we identified 168 candidate direct NMD targets *in vivo*. Remarkably, we found that 81% of direct target genes do not show increased expression levels in an NMD mutant, presumably due to feedback regulation. Because most previous studies have used up-regulation of mRNA expression as the only means to identify NMD-regulated transcripts, our results provide new directions for understanding the roles of the NMD pathway in endogenous gene regulation during animal development and physiology. For instance, we show clearly that direct target genes have longer 3′ untranslated regions compared with nontargets, suggesting long 3′ untranslated regions target mRNAs for NMD *in vivo*. In addition, we investigated the role of NMD in suppressing transcriptional noise and found that although the transposable element *Copia* is up-regulated in NMD mutants, this effect appears to be indirect.

Steady-state messenger RNA (mRNA) expression levels are controlled by a balance of *de novo* transcription and transcript degradation. Nonsense-mediated mRNA decay (NMD) is a degradation mechanism that functions to target mRNAs in a translation-dependent manner (Kervestin and Jacobson 2012). The machinery required to execute NMD is evolutionarily conserved (Behm-Ansmant *et al.* 2007b), with three core proteins, Upf1, Upf2, and Upf3, found throughout the eukaryotes. Four other well-characterized NMD factors, Smg1, Smg6, and the paralogous proteins Smg5 and Smg7, are thought to function as regulators of the core components and are required for NMD in many, but not all organisms (Behm-Ansmant *et al.* 2007b; Riehs *et al.* 2008; Lloyd and Davies 2013). NMD function is required for viability in complex organisms, including *Drosophila* (Metzstein and Krasnow 2006), zebrafish (Wittkopp *et al.* 2009), mammals (Medghalchi *et al.* 2001; McIlwain *et al.* 2010; Weischenfeldt *et al.* 2012), and plants (Kerényi *et al.* 2008). In contrast, in simpler organisms, including *Saccharomyces cerevisiae* (Leeds *et al.* 1991), *Schizosaccharomyces pombe* (Mendell *et al.* 2000), and *Caenorhabditis elegans* (Hodgkin *et al.* 1989), NMD is not required for viability. However, loss of NMD pathway function leads to stress sensitivity in yeasts (Leeds *et al.* 1991; Rodríguez-Gabriel *et al.* 2006) and abnormal morphogenesis in *C. elegans* (Hodgkin *et al.* 1989), indicating that NMD does have important biological roles in these organisms. Loss-of-function mutations in different NMD genes lead to a similar spectrum of defects within an organism (Hodgkin *et al.* 1989; Rehwinkel *et al.* 2005; Metzstein and Krasnow 2006; Wittkopp *et al.* 2009; Avery *et al.* 2011; Frizzell *et al.* 2012), suggesting that, although a number of NMD pathway components may function in NMD-independent processes (Azzalin and Lingner 2006; Roberts *et al.* 2013), NMD is in itself required for proper development and physiology.

It is not known why organisms rely on a functional NMD pathway for viability. The best-known targets of the NMD pathway are transcripts harboring premature termination codons (PTCs). One possibility is that all organisms contain a background load of PTC-containing transcripts, arising from inheritance or by sporadic errors in transcription or splicing, and that the NMD pathway is required to eliminate these erroneous mRNAs to maintain normal cellular function (Neu-Yilik *et al.* 2004). An alternative model is that NMD regulates a set of endogenous, error-free transcripts and this regulation is required for development and viability. One observation in support of this latter model is that loss of NMD pathway function results in changes in the expression levels of many endogenous genes (He *et al.* 2003; Mendell *et al.* 2004; Rehwinkel *et al.* 2005; Metzstein and Krasnow 2006; Weischenfeldt *et al.* 2008; Ramani *et al.* 2009; Wittkopp *et al.* 2009; Yepiskoposyan *et al.* 2011; Rayson *et al.* 2012). These studies have found that up to 15% of all genes in diverse organisms may be under constitutive negative regulation by the NMD pathway. On the basis of these analyses, a model has emerged wherein NMD-mutant phenotypes, including lethality, are due to misregulation of specific native targets (Hwang and Maquat 2011; Palacios 2013). However, it is unclear how many genes that are misregulated in NMD mutants are directly targeted by the NMD pathway *vs.* those genes that are indirectly targeted, and which direct NMD targets are responsible for the organismal defects observed in NMD mutants. Distinguishing between direct and indirect targets is thus important to understanding NMD mutant phenotypes, as well as the mechanisms by which the NMD machinery recognizes endogenous substrates.

In a few studies, investigators have sought to identify direct NMD targets by using a number of different approaches. Splicing-sensitive microarrays have been used to identify instances of differential change in expression of specific mRNA isoforms when NMD is inhibited (Barberan-Soler *et al.* 2009; Hansen *et al.* 2009). Because changes in transcription after NMD disruption are expected to affect all splice isoforms equally, differential NMD-sensitivity of different splice isoforms indicates direct targeting. However, NMD has been shown to functionally regulate splicing factors (Barberan-Soler *et al.* 2009; Weischenfeldt *et al.* 2012), so relative changes in isoform expression may not only be due to direct targeting. Another approach to assess direct NMD targeting is to identify which RNAs are bound to the critical NMD factor Upf1, because direct NMD targets are known to show enriched Upf1 binding (Johansson *et al.* 2007; Hurt *et al.* 2013; Matia-González *et al.* 2013). Such binding does not, however, unequivocally identify NMD substrates, as Upf1 also binds many non-NMD targets (Hogg and Goff 2010; Zünd *et al.* 2013).

Another genome-wide technique that can be used to identify NMD targets is analysis of mRNA decay rates, with the assumption that direct NMD targets will show increased stability upon inhibition of NMD. Using such an approach, Guan *et al.* (2006) compared rates of mRNA decay between *Upf1^+^* and *Upf1*Δ yeast strains after treatment with the transcriptional inhibitor thiolutin. This analysis revealed that 220 of 616 genes (36%) that are up-regulated in *Upf1*Δ mutants had slower decay than that observed in the *Upf1*^+^ background. Recently, Tani *et al.* (2012a) globally measured mRNA decay rates in HeLa cells depleted of *Upf1* by siRNA knockdown. In this case, the authors used the RNA sequencing-based BRIC-seq technique (Tani *et al.* 2012b), which allows for monitoring of decay without the use of transcriptional inhibitors. They found that the mRNAs of 76 of 324 genes (23%) that were significantly up-regulated after *Upf1* knockdown were stabilized in the absence of *Upf1*. Thus, both these studies conclude that the majority of genes up-regulated in the absence of a functional NMD pathway are not directly targeted by the NMD machinery.

A final approach that has been used to identify direct NMD-pathway substrates is the use of an NMD “reactivation” assay (Maderazo *et al.* 2003; Johansson *et al.* 2007). This assay, developed in yeast cells, uses an inducible system to rapidly restore levels of an NMD gene in a cognate NMD mutant background. Before reactivation, levels of both direct and indirect NMD-regulated genes accumulate. After reactivation, direct targets are expected to be rapidly degraded whereas levels of secondary targets will decrease only after direct target levels are normalized. Thus, by monitoring mRNA levels over a reactivation time course, direct and secondary NMD targets can be distinguished. Using this approach in *S. cerevisiae*, Johansson *et al.* (2007) found that 427 of 792 (54%) of genes that are overexpressed in an NMD mutant appear to be direct targets, as defined by the reactivation assay, and conversely, 68% of direct targets are overexpressed in NMD mutants.

No study has yet been performed to experimentally distinguish direct from indirect NMD targets in an intact, multicellular organism. Here, we describe the use of larval *Drosophila melanogaster* to characterize NMD targeting in such a setting. First, we used RNA sequencing to identify transcripts with altered expression levels in animals mutant for the NMD gene *Upf2*. Next, to identify direct targets, we adapted the yeast NMD reactivation assay so as to distinguish rapidly degrading direct targets from slower responding indirect targets. To this end, we reintroduced *Upf2* into *Upf2*-deficient larvae and identified transcripts that were depleted rapidly on a genome-wide scale. We find that a minority of genes up-regulated in an NMD mutant appear to be direct targets. Strikingly, we also find that a majority of directly targeted genes do not show increased expression at steady state when the NMD pathway is inactivated. Bioinformatic analysis of our candidate direct targets reveals that these genes have on average significantly longer 3′ untranslated regions (UTRs) then nontarget control transcripts, suggesting that a long 3′ UTR is a primary mechanism of NMD targeting in intact *Drosophila*, as has been proposed based on cell culture experiments (Behm-Ansmant *et al.* 2007a). We also use our reactivation assay to demonstrate that an observed increase in retrotransposon expression, known to occur in NMD mutants, is likely due to indirect effects of NMD pathway disruption.

## Materials and Methods

### Fly stocks and genetics

All fly stocks were reared according to standard protocols (Sullivan *et al.* 2000). Balancers used were *FM7i*, *Act5C:GFP*, and *FM7c*. The mutant NMD alleles are described in Metzstein and Krasnow (2006). *Upf2^25G^* is on the chromosome *y w Upf2^25G^ FRT^19A^* and for a control, we used *y w FRT^19A^* (Xu and Rubin 1993). The heat-shock GAL4 is w*; *P{w[+mC]=GAL4-Hsp70.PB}89-2-1* (Bloomington *Drosophila* Stock Center #1799) (Roman *et al.* 2000).

### Construction of the *UAS-Upf2* transgene

The long *Upf2* coding sequence plus 3′ UTR (4767 bp) required a multistep procedure to generate the full-length rescuing transgene. First, we used the primer pairs Upf2xF1 and Upf2xR1 to amplify a 140 bp 5′ fragment and the primer pair Upf2xF2 and Upf2xR2 to amplify a 500 bp 3′ fragment at the end of the *Upf2* 3′ UTR, using the full-length *Upf2* cDNA RE04053 (Stapleton *et al.* 2002) as a template. The two fragments were then joined (based on overlapping sequences present in Upf2xR1 and Upf2xF2) using Upf2xF1 and Upf2xR2. The resulting product was TOPO-TA cloned in pCR4 (Invitrogen, Carlsbad, CA) and sequenced to confirm that no errors were introduced during polymerase chain reaction (PCR) amplification. We then replaced the middle section of this clone (using naturally occurring *Nhe*I and *Bst*BI restriction sites present in the *Upf2* gene) with a 4.3-kb *Nhe*I/*Bst*BI fragment from RE04053. Finally, we subcloned this full-length cDNA using *Not*I and *Sma*I sites present in the primers, into *Not*I/*Stu*I-digested pUAST (Brand and Perrimon 1993). Note, this procedure removes the SV40 3′ UTR present in pUAST, which is itself sensitive to NMD-mediated degradation (Metzstein and Krasnow 2006). Flies were transformed with the pUAST construct using standard *P*-element−mediated transgenesis. Transgenic injections were performed by BestGene Inc. (Chino Hills, CA). Primer sequences listed in Table S4.

### RNA isolation and cDNA preparation

Whole animals or cells were homogenized in Trizol (Invitrogen) and total RNA extracted using a standard three-phase chloroform technique (Chomczynski and Sacchi 1987). RNA was purified and concentrated using the RNeasy column (QIAGEN Inc., Gaithersburg, MD), including an on-column treatment with DNase I. Random decamer-primed cDNA was prepared using M-MLV reverse transcriptase (RNase H+) as part of the RETROscript kit (Ambion, Austin, TX). For each reverse transcription reaction, 10 μg of input RNA was used to aid in comparisons of absolute abundance between samples.

### RNA sequencing

For each replicate (two control and two mutant), we collected 11 male larvae of genotype *y w FRT^19A^/Y* (control) or *y w Upf2^25G^ /Y* (mutant) in a 0- to 4-hr time period past the L2/L3 molt. Male larvae where identified by sorting using the *FM7i*, *Act5C:GFP* balancer (for *Upf2^25G^*), or by morphological criteria (for the control). Total RNA was isolated using Trizol/chloroform and quality assessed using a 2100 Bioanalyzer (Agilent, Santa Clara, CA). cDNA synthesis and sequencing was performed by Cofactor Genomics (St. Louis, MO). In brief, rRNA was removed from the sample using RiboMinus Eukaryote Kit (Invitrogen), 1 µg of this purified RNA was fragmented using reagents in the Illumina RNA-seq kit (Illumina, San Diego, CA), then reverse transcribed using random hexamers and Superscript II (Invitrogen, Carlsbad, CA), and the second strand synthesized with DNA Pol I and RNase H. To construct a sequencing DNA library, the double-stranded cDNA was blunted, tailed with an A base, ligated with paired-end adaptors in the Illumina RNA-seq kit (Illumina, San Diego, CA), and size selected on a polyacrylamide gel. Sequencing was performed on an Illumina GAIIx platform, according to manufacturers protocols.

### Quantification of RNA-seq data

We used the USeq package to identify differentially expressed genes (Nix *et al.* 2008). In brief, USeq counts the number of reads aligned to each annotated gene and uses the DESeq algorithm to prioritize genes according to their *P*-values of being differentially expressed (Anders and Huber 2010). Internally, a negative binomial distribution is adopted by DESeq to model the discrete read counts in each sample and account for the overdispersion of the coverage data.

### Reactivation and microarray experiments

RNA was isolated, as described previously, from the following genotypes: *Upf2^25G^/FM7i*, *Act5C:GFP*; *UAS-Upf2/hsp70-GAL4* (experimental), and *Upf2^25G^/FM7i*, *Act5C:GFP*; *hsp70-GAL4/+* (control). Library construction and hybridization were performed at the GNomEx core at the Huntsman Cancer Institute at the University of Utah. cDNA was generated in a poly dT-primed reverse-transcription (RT)-PCR using MMLV-RT. Labeled cRNA was generated by incorporating cyanine 3-CTP or cyanine 5-CTP into a T7 RNA polymerase reaction using the cDNA as template. Hybridization was performed on an Agilent *Drosophila* 44K array using a Agilent SureHyb hybridization oven. Hybridized chips were scanned using a Agilent G2505C Microarray Scanner and signal processing accomplished with Agilent Feature Extraction software, v 10.5. Stepwise normalization was performed with the Limma package in R (Smyth and Speed 2003), including background signal correction, normalization within microarrays, and normalization between microarrays.

### Identification of reactivation targets

To identify high confidence reactivation targets, we first applied a number of filtering steps to the data obtained from our microarray time course. We first eliminated genes from the analysis that displayed expression changes of more than 20% from the preheat shock time point to the 4-hr time point in control experiment (without cDNA). This was found to be primarily due to low absolute expression levels. Next, we verified that leaky expression from the *Upf2*:cDNA transgene did not lead to rescue of NMD target gene levels by comparing expression between the experimental and control samples at the prior-to-heat shock time point, and removed any genes that differed in expression by more than 50% between the two condition. In this analysis, we did not observe consistent rescue of target genes. Finally, those genes which did not have high-confidence annotations in FlyBase were also removed.

We then used criteria similar to those used by Johansson *et al.* (2007) to identity reactivation targets in the filtered set. Our primary criterion was a decrease in expression of at least 1.8-fold over the 4-hr time course in the animals carrying the rescue cDNA and that this decrease in expression differed significantly from control animals, which were heat shocked but did not carry the rescuing cDNA. To perform this latter analysis, we first converted each of the four time points in the experiment to a nominal ordinal value: the time point before the heat shock is 1; the time point right after the heat shock is 2; and so on. For each gene represented on the array, we calculated the Pearson correlation coefficient between its expression level at each time point and the ordinal time value. Permutation tests were used to evaluate the significance of association between the expression and the time of treatment. Genes in this regression analysis that had negative slopes and differed significantly from control (*P* < 0.1), as well as the 1.8-fold decrease from the preheat shock time point, were selected as reactivation targets. Genes that did not fulfill either of these criteria were defined as our control, nondirect target set.

### Statistical analysis

We performed logistic regressions to evaluate the relationship between NMD targeting and several features by using the reactivation target set and nonreactivation target set. Tested transcript features included predictor variables: 3′ UTR length; 3′ UTR RNA structure, as evaluated by CentroidFold RNA folding energy (Hamada *et al.* 2009); the presence of introns in 3′ UTR; potential stop-codon read-through events, as defined by (Jungreis *et al.* 2011); the length ratio between 3′ UTR and the coding sequence; the presence of bi/poly-cistronic transcripts; and the presence of stop codons in the 3′ UTR. For qualitative features, we set nominal values for the presence or absence of the feature.

### Amplicon design and quantitative RT-PCR (qRT-PCR)

Primer3 (http://frodo.wi.mit.edu/) was used to design amplicons for qRT-PCR experiments using a primer size of 23 bp, a melting temperature of 62°, and designed to amplify a region of 60−100 bps. When possible, the amplicon, or the primers themselves, was designed to span splice junctions to minimize the amplification of contaminating genomic DNA. Primer pairs were tested for amplification efficiency with a wild-type cDNA dilution series. The following primers were used: *Gadd45* (1: qGadd45_F1 and qGadd45_R1; 2: qGadd45_F2 and qGadd45_R2); *RP49*: qRP49_F and qRP49_R; *Copia*; qCopia_F and qCopia_R; and *tra*: qtra_F and qtra_R. All qRT-PCR analysis was conducted using iQ SYBR Green master mix and a MyiQ thermal cycler running iQ5 v2.0 software (Bio-Rad, Hercules, CA). *RP49* (*RpL32*) served as a reference gene in all cases. Primer sequences listed in Table S4.

### S2 cell culture and RNA interference (RNAi)

We cultured *Drosophila* S2 cells at room temperature in Schneider’s media (Invitrogen) supplemented with 10% fetal bovine serum and antibiotics. To deplete cells of Upf1, we amplified a 771 base pair region from the coding sequence of *Upf1* using primers with T7 RNA polymerase sites at the 5′ ends (Upf1_RNAi_F and Upf1_RNAi_R), and used this product to synthesize double-stranded RNA (Megascript kit; Ambion). We then incubated S2 cells with this dsRNA in serum-free media for 45 min, replaced the serum, and allowed the cells to recover for 3 d. We retreated with dsRNA to maintain the knockdown, allowed a 3-d recovery, and then mock-treated or treated the cells with actinomycin D for 1 hr. RNA was collected and purified using Trizol reagent (Invitrogen) and used to synthesize cDNA for qRT-PCR analysis.

## Results

### Characterization of *Upf2^25G^* mutant larvae

**Figure 1:**
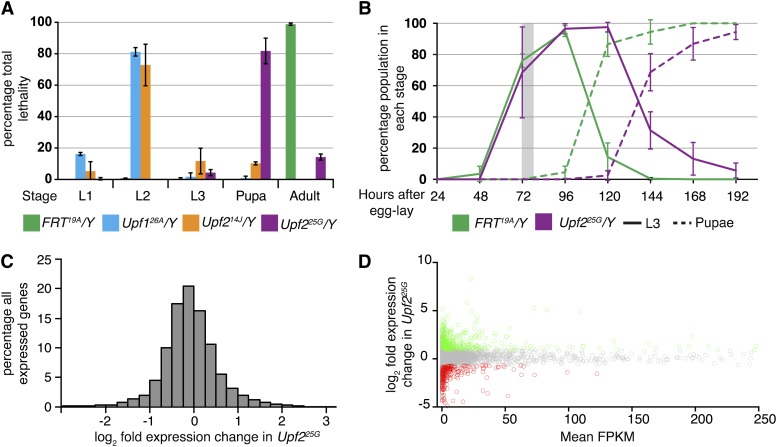
Phenotypic and transcriptome analysis of *Upf2^25G^*. (A) Effective lethal phase of NMD mutants. Null mutations in *Upf1* (*26A*; cyan) and *Upf2* (*14J*; orange) result in death during the second larval instar, as compared with control (green), the great majority of which survive to adulthood. Animals hemizygous for a hypomorphic allele of *Upf2* (*25G*; purple) die mostly as pupae, with approximately 14% escaping into adulthood. (B) *Upf2^25G^ /Y* animals develop at a normal rate into L3 but are delayed in this stage of larval development. Solid lines represent the proportion of animals in the L3 stage at each time point; dashed lines represent the proportion in pupal stages. The gray box indicates the collection window (0- to 4–hr-old L3s) in which we compared gene expression changes between control and *Upf2^25G^/Y*. (C) Proportion of genes based on relative expression in *Upf2^25G^/Y* compared with control, based on normalized RNA-seq read depth. (D) Scatter plot of all genes (gray circles) with their average expression level on the x-axis and their relative expression change in *Upf2^25G^* mutants on the y-axis. Significantly up-regulated and down-regulated genes (*P* < 0.01) are represented by green and red circles, respectively. In (A) and (B), error bars represent ± 1 SD (n > 118 for all genotypes).

Previously, we have shown that hemizygous males harboring a strong hypomorphic mutation in *Upf2*, called *25G*, leads to stabilization of NMD-sensitive reporter mRNAs, PTC bearing mutant transcripts, and endogenous targets of NMD (Metzstein and Krasnow 2006). However, we found that, unlike null alleles of *Upf1* or *Upf2*, which impart lethality in the second larval stage (L2), the development of *Upf2^25G^/Y* animals proceeds normally through these stages (Figure 1A). The start of the third larval stage (L3) also begins normally in *Upf2^25G^*/*Y*, but development then slows such that progression into the pupal stage is delayed by approximately 24 hr (Figure 1B). Most *Upf2^25G^/Y* die as pupae, with only 14% of animals eclosing into adulthood (Figure 1A). Using the *25G* hypomorphic mutation at the early L3 stage thus allows us to examine the effects of NMD pathway disruption without the complications of lethality and developmental delay inherent to *Upf2* null mutants.

### Analysis of the transcriptome of *Upf2^25G^* mutant larvae

To analyze the effect of NMD pathway disruption on endogenous gene expression, we performed deep-sequencing on total RNA extracted from *Upf2^+^/Y* and *Upf2^25G^/Y* L3 larvae. Control and mutant males were stage matched by rearing to 0−4 hr after the L2-L3 larval molt, and total RNA was extracted and sequenced on the GAx II Illumina platform (see *Materials and Methods*). Sequencing reads were aligned to the reference genome (dm3) using Tophat software under the pair-end mode (Trapnell *et al.* 2012). We analyzed two biological replicates each of mutant and control samples and were able to align 24−53 million read pairs to the genome per sample (Supporting Information, Table S1). Although we found reads representing expression of 14,699 genes, only 10,434 were detected in all four samples and so this subset was used for subsequent statistical analysis (File S1). Of these 10,434 genes, we identified 413 (4.0%) as being differentially up-regulated at a significance of *P* < 0.01 (Figure 1, C and D; top genes listed in Table S2 and all genes listed in File S2). These genes were up-regulated between 1.8- and 330-fold in *Upf2^25G^* compared with control samples. A total of 247 genes were found to be down-regulated in *Upf2^25G^/Y* (Table S2 and File S2). We found that genes encompassing a broad range of absolute expression levels are subjected to NMD-mediated regulation (Figure 1D, Table S2, and File S2).

### Identification of NMD reactivation targets

**Figure 2:**
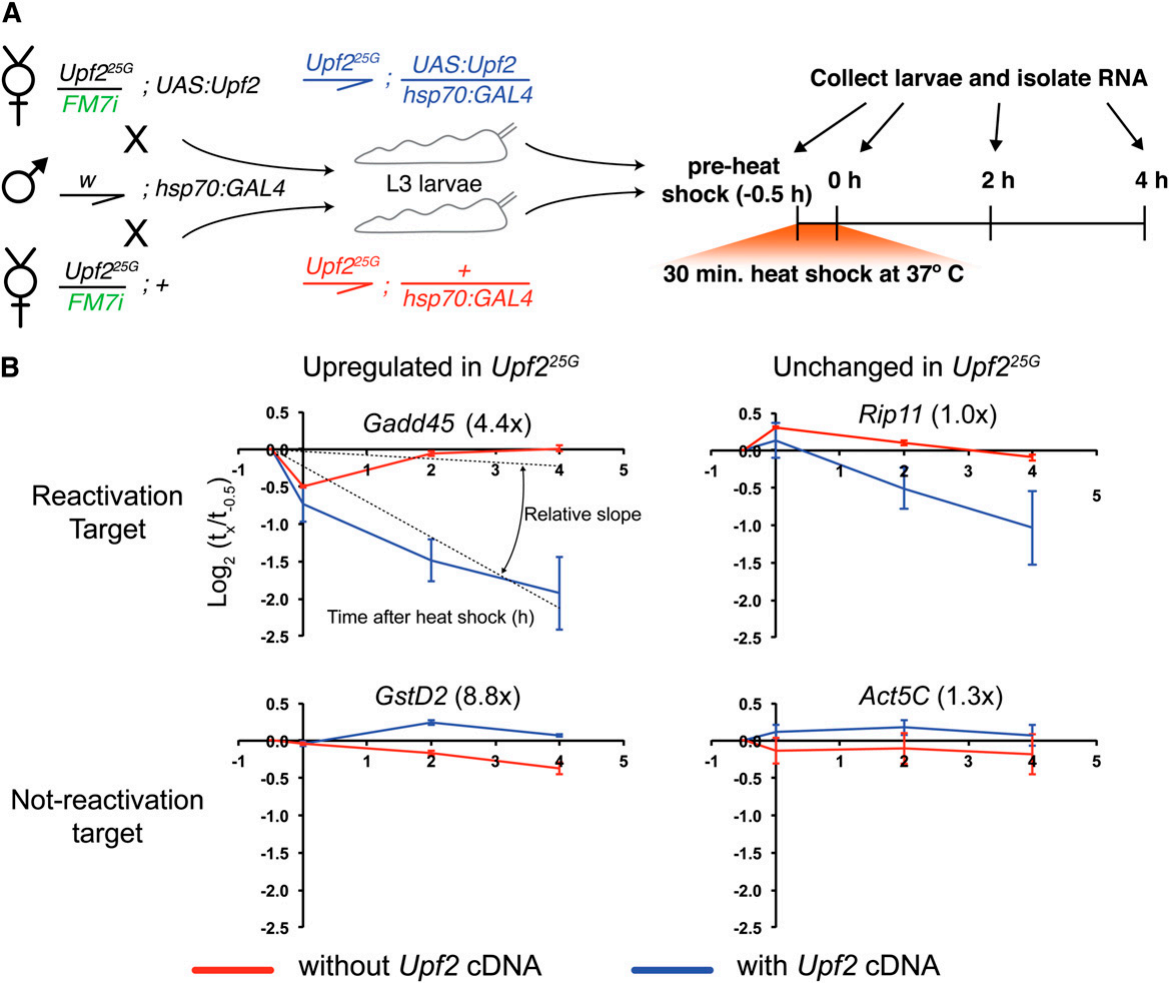
Identification of direct targets of NMD using reactivation of *Upf2*. (A) Crosses used to generate larvae. *Upf2^25G^/FM7i* ; *UAS:Upf2* or *Upf2^25G^/FM7i* females were crossed to *w/Y*; *hsp70:GAL4* males. *Upf2^25G^/Y* ; *UAS:Upf2/ hsp70:GAL4* (experimental, blue) or *Upf2^25G^/Y* ; *+/ hsp70:GAL4* (control, red) L3 male larvae were collected based on *Act5C:GFP* carried on the *FM7i* balancer (represented in green) and male gonadal morphology. Larvae of the appropriate genotype were subjected to the heat-shock regime indicated on the right. (B) Examples of genes in the four classes identified; *Gadd45* (up-regulated in *Upf2^25G^/Y*, reactivation target; upper left); *Rip11* (not up-regulated in *Upf2^25G^/Y*, reactivation target; upper right); *GstD2* (up-regulated, nonreactivation target; lower left); *Actin5C* (not up-regulated, nonreactivation target). Numbers in parentheses represent the fold change observed in *Upf2^25G^/Y* compared with control. X-axis represents the time points collected as in (A). Y-axis is relative expression level (on a log_2_ scale) compared to the pre-heat shock time point (−0.5 hr) within each genotype. We also define a relative decay slope for each condition, calculated from the regression line of log-transformed data throughout the time course, equal to the slope of the experimental condition (with cDNA) minus the slope of the control condition (without cDNA). Error bars represent ± 1 SD.

To identify which transcripts are directly targeted by the NMD pathway, we used a pathway reactivation assay. Starting with an NMD mutant, in which expression levels of both direct and indirect targets should be elevated, a wild-type copy of the mutated factor is expressed, and RNA expression levels are measured over time. It is expected that expression levels of transcripts that are direct targets of NMD should decrease rapidly. Conversely, the levels of indirect targets will remain high until direct targets return to wild-type levels. To perform this experiment, we used the bipartite *UAS/GAL4* expression system (Brand and Perrimon 1993) to rapidly restore wild-type *Upf2* from a *UAS:Upf2* transgene in *Upf2^25G^/Y* mutant L3 larvae by using the *hsp70* enhancer to drive GAL4 in a heat-inducible manner (Figure 2A). We confirmed that the *UAS:Upf2* transgene was functional by testing for rescue; we found that *UAS:Upf2* driven by *Act5C:GAL4* could rescue the null allele *Upf2^14J^* to full viability in hemizygous males (data not shown). In addition, we observed no lethality or other defects from driving high-level expression of *Upf2*, either constitutively, using *Act5C:GAL4*, or acutely after heat shock with *hsp70:GAL4*, suggesting that these experimental conditions are unlikely to result in ectopic degradation of non-NMD targets.

Our time course consisted of a preheat-shock time point, a t = 0 time point collected directly after a 30-min heat shock, and two further time points, 2 and 4 hr after the completion of the heat shock. As a control for the effect of the heat shock itself on gene expression, we analyzed larvae that harbored *Upf2^25G^* and heat-shock GAL4 but not the *UAS:Upf2* transgene. These larvae were treated to the same heat-shock regime as the experimental samples. At each of the time points, for both experimental and control samples, RNA was collected and expression levels were measured using microarrays (see *Materials and Methods*). Analysis of *Upf2* expression levels revealed that *Upf2* was activated over our time course approximately 12-fold in experimental animals, but remained unchanged in control animals, as expected (Figure S1).

We next developed a statistical model to test whether, for each gene, reactivation of the NMD pathway led to a change in mRNA abundance. Our primary assumption was that the expression levels of mRNAs that are direct targets of the NMD pathway would decrease rapidly during the course of the experiment. We defined two parameters to define whether a gene was a direct NMD target. First, the expression level at the end of the 4-hr time course had to decrease by 1.8-fold compared with preheat shock levels. Second, changes in expression were compared with control samples (heat shocked, but lacking the *UAS:Upf2* transgene) to control for effects of heat shock and culturing conditions, with a negative binomial model used to compare differences in gene expression at each time point (see *Materials and Methods*). The difference in stability between experimental and control conditions is also described by a best-fit linear regression slope of the log-transformed expression level throughout the time course, which assumes exponential decay of target genes. We refer to this difference in stability as “relative slope” (Figure 2B).

After eliminating transcripts that displayed inconsistent expression levels throughout the time course, such as those that were not expressed at high enough levels for meaningful analysis (see *Materials and Methods* for details), we were left with 6956 genes (File S3). We found a small number of genes (154) that displayed the rapid decrease in expression levels in experimental samples compared to controls defined above, and are thus putative direct targets of the NMD pathway (File S4). We refer to these genes as reactivation targets. The other 6802 genes are defined as nonreactivation targets and are not likely to be directly regulated by the NMD pathway, although we cannot rule out the possibility that some of these are indeed targets but their expression does not change significantly enough to be identified under our experimental conditions.

Among the reactivation targets, we found well-known direct NMD targets, including the NMD pathway components *Smg5* and *Smg6*, whose homologs are direct NMD targets in mammals (Huang *et al.* 2011; Yepiskoposyan *et al.* 2011), and have also been shown to be direct targets in *Drosophila* cell culture (Rehwinkel *et al.* 2005). We also found as a target *Gadd45*, homologs of which are known to be direct NMD targets in mammals (Viegas *et al.* 2007; Huang *et al.* 2011; Tani *et al.* 2012a). To validate that other reactivation targets represent direct substrates of the NMD pathway, we directly measured transcript stability of several of them in *Drosophila* S2 cells with and without a functional NMD pathway. Depletion of *Upf1* by RNAi resulted in stabilization of these mRNAs (Figure S2), indicating our reactivation targets likely represent genuine direct targets of NMD. No particular gene ontology category was enriched in the reactivation data set, implying that NMD may regulate a functionally diverse array of target genes.

### Comparison of mutant expression changes and reactivation targets

By combining the expression data from the RNA-seq experiment with our reactivation experiment, we assigned genes to one of four classes (Figure 2B). Some genes, such as *Gadd45*, are up-regulated in *Upf2^25G^* and were identified as reactivation targets. Others, such as *GstD2*, are up-regulated in mutant animals but did not rapidly decay after reactivation of *Upf2*. Conversely, some genes, such as *Rip11*, are not overexpressed in *Upf2^25G^*, but still had mRNA levels that decreased rapidly upon *Upf2* reactivation. Finally, most genes are neither up-regulated in *Upf2^25G^* nor change during the reactivation experiment. This latter class includes genes such as *Act5C* that are known be unaffected by NMD in numerous experiments.

**Figure 3:**
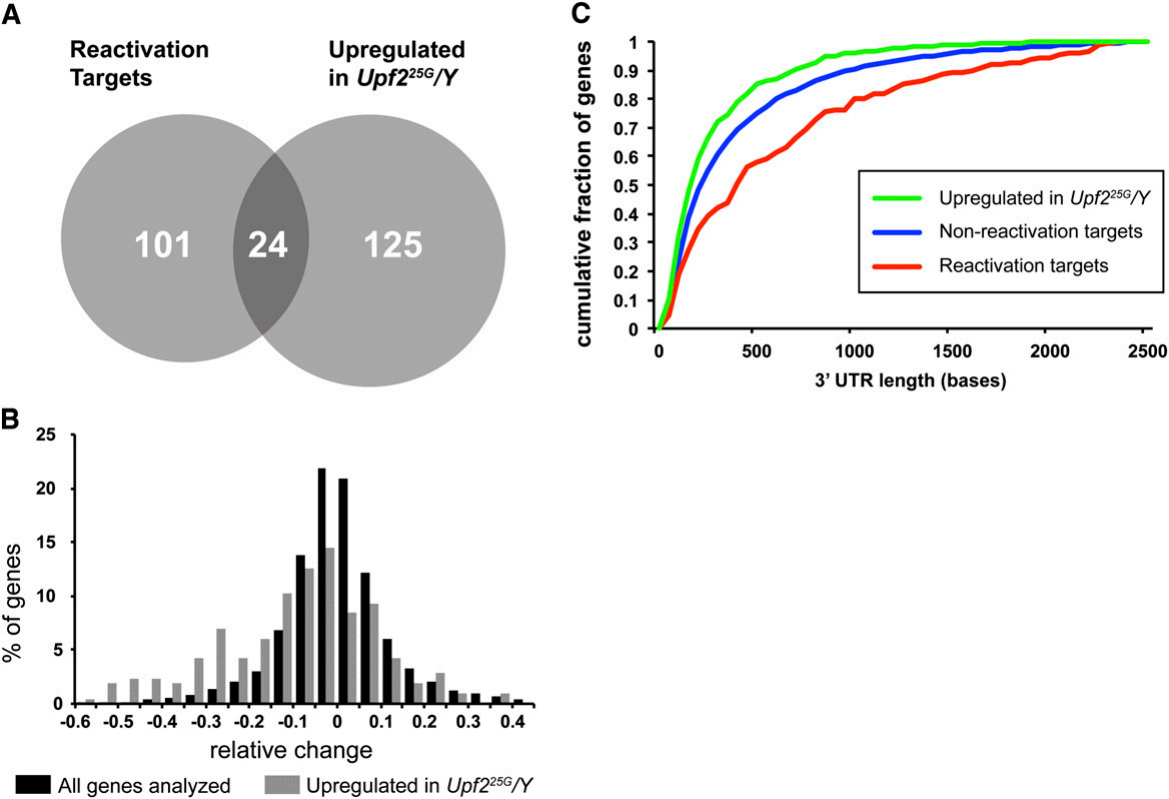
Analysis of reactivation targets. (A) Venn diagram showing overlap of genes that display increased expression in *Upf2^25G^/Y* and reactivation targets. (B) Distribution of relative change in slope (defined in Figure 2) identified in reactivation experiment for all genes (black bars) and genes up-regulated in *Upf2^25G^/Y* (gray bars). (C) Cumulative percentage of 3′ UTR lengths measured in nonreactivation targets genes (5291 genes; blue), reactivation targets (168 genes; red), and genes up-regulated in *Upf2^25G^/Y* (214 genes; green).

To compare the sets of genes with expression changes in *Upf2^25G^* mutants with reactivation targets, we restricted our analysis to genes that were both represented on our microarray, present in our deep sequencing data set, and were well annotated in FlyBase (see *Materials and Methods*). A total of 5539 genes fulfilled these criteria (File S5), of which 149 are up-regulated in *Upf2^25G^* and 125 are reactivation targets. Common to both the up-regulated and reactivation target sets were 24 genes. Therefore, of the 149 up-regulated genes, we found only 16% (24/149) are reactivation targets (Figure 3A and Table 1). These data suggest that 84% of genes up-regulated in the *Upf2^25G^* mutant are indirect targets of the NMD pathway, likely responding to expression changes of direct targets. However, that 16% of genes up-regulated in NMD mutants are found to be reactivation targets represents a significant enrichment over the entire analyzed gene set, in which only 2.3% (125 of 5539) of genes are reactivation targets (Fisher exact test *P*-value = 5.85 × 10^−12^). In addition, the relative slope of the 149 up-regulated genes in *Upf2^25G^* mutants is skewed negatively, corresponding to degradation during NMD reactivation (Figure 3B, gray bars), compared with the distribution across all 5,539 genes in the analysis (Figure 3B, black bars), also indicating that up-regulated genes are enriched for direct targets.

**Table 1:** The 24 genes up-regulated in *Upf2^25G^* and identified as reactivation targets, sorted by increased expression observed in mutant

FBgn	Gene Name	Fold Up-Regulation in *Upf2^25G^*	*P* Value	Relative Slope	Relative Slope *P*-Value	Fold Decay-4 hr
FBgn0040837	*CG8620*	11.84	2.38E-13	−0.26	0.039	2.44
FBgn0034605	*CG15661*	8.84	1.39E-03	−0.43	0.082	2.20
FBgn0033240	*CG2906*	7.30	1.95E-07	−0.08	0.043	1.80
FBgn0029766	*CG15784*	6.52	1.29E-16	−0.40	0.040	2.42
FBgn0261113	*Xrp1*	6.52	3.51E-17	−0.39	0.041	1.94
FBgn0019890	*Smg5*	5.33	5.90E-05	−0.54	0.044	2.44
FBgn0033153	*Gadd45*	5.00	2.23E-06	−0.70	0.040	4.82
FBgn0039319	*CG13659*	4.85	1.84E-10	0.00	0.081	2.37
FBgn0037936	*CG6908*	4.50	1.18E-11	−0.68	0.039	5.21
FBgn0034501	*CG13868*	4.33	9.15E-11	−0.34	0.042	2.12
FBgn0014031	*Spat*	4.19	2.24E-10	−0.15	0.087	2.68
FBgn0031643	*CG3008*	3.94	4.97E-09	−0.14	0.043	1.85
FBgn0041627	*Ku80*	3.58	1.49E-05	−0.26	0.040	1.85
FBgn0032981	*CG3635*	3.34	4.00E-03	−0.19	0.077	2.06
FBgn0037391	*CG2017*	3.34	1.11E-04	−0.20	0.034	2.32
FBgn0050424	*CG30424*	3.31	1.11E-03	−0.46	0.082	2.34
FBgn0039328	*CHKov2*	3.14	3.11E-05	−0.44	0.036	2.66
FBgn0039260	*Smg6*	2.88	3.13E-05	−0.46	0.039	2.07
FBgn0035476	*CG12766*	2.70	1.47E-04	−0.54	0.076	2.16
FBgn0042105	*CG18748*	2.60	3.14E-03	−0.72	0.075	7.18
FBgn0086370	*sra*	2.59	8.02E-05	−0.11	0.082	1.87
FBgn0037781	*Fancl*	2.51	1.58E-03	−0.23	0.083	2.31
FBgn0085194	*CG34165*	2.13	3.54E-03	−0.11	0.049	1.81
FBgn0039226	*Ude*	1.91	7.12E-03	−0.50	0.088	2.20

Fold up-regulation and associated *P* value are obtained from RNA-seq data. “Relative slope” refers to the decay of transcript in *Upf2*-reactivated larvae calculated as a linear regression of log_2_ transformed expression level relative to control larvae; see Figure 2. “Fold decay-4 hr” refers to the fold change in transcript level between the preheat shock and 4-hr time point in animals carrying the *Upf2* cDNA. FBgn, FlyBase gene number.

Most remarkably, these data indicate that the majority (125 of 149) of reactivation targets are not increased at steady state in *Upf2^25G^* (Figure 3A). Thus, even though these genes appear to be direct targets, their steady expression levels are unaffected by loss of NMD pathway function.

### Structural features of genes regulated by the NMD pathway

Several kinds of sequence and structural features are thought to influence whether an mRNA is targeted by NMD (Schweingruber *et al.* 2013). Current models of NMD, particularly in nonvertebrates, suggest 3′ UTR length is a primary signal that stimulates NMD targeting (Kebaara and Atkin 2009; Kervestin and Jacobson 2012). Other features that may direct transcripts for degradation include a weakly structured 3′ UTR (Eberle *et al.* 2008); a low ratio of coding sequence to 3′ UTR length (Brogna and Wen 2009); the presence of more than one open reading frame in the transcript, including small upstream open reading frames (He *et al.* 2003); the presence of introns in the 3′ UTR (Le Hir *et al.* 2000); and the use of stop codon read-through as a regulatory mechanism (Jungreis *et al.* 2011). Therefore, we asked whether any of these features are enriched in our NMD reactivation targets compared with nontargets (Table 2). Most strikingly, we found the median length of the 3′ UTR in reactivation targets was 423 bases, considerably longer than the 218 bases found in the nonreactivation control set (*P* = 1.55 × 10^−5^ from regression analysis) (Figure 3C). We did not detect significant enrichment for presence of introns in the 3′ UTR, the ratio of length of the coding sequence to 3′ UTR, predicted free energy of the 3′ UTR, read-through candidates, stop codon density in the 3′ UTR, or polycistronic transcripts (Table 2). In contrast to reactivation targets, the 149 mRNAs that show increased expression in the *Upf2^25G^* mutant have significantly shorter 3′ UTRs on average than genes in our control set (median of 159 bases, *P* = 8.71 × 10^−8^; Table 2 and Figure 3C).

**Table 2:** Statistical comparison of NMD-sensitive genes compared with controls

	Nonresponding Genes*^a^*	Up-Regulated in *Upf2^25G^**^c^*		Reactivation Targets*^e^*		Up-Regulated Reactivation Targets*^f^*	
	Count or Average*^b^*	Count or Average	*P* Value*^d^*	Count or Average	*P* Value	Count or Average	*P* Value
Total	5289	215	n/a	154	n/a	24	
3′ UTR length, bases	450.2	275.4	8.7E-08	739.6	1.6E-05	261.4	2.8E-01
3′ UTR structure, kcal/mol/base	−0.024	−0.023	0.1006	−0.028	0.772	−0.028	0.161
CDS/3′ UTR length ratio	0.416	0.389	0.200	0.661	0.601	0.301	0.990
Read-through candidates	113	1	0.358	5	0.700	0	0.520
Bicistronic genes	23	2	6.5E-02	0	0.570	0	1.000
3′ UTR intron present	256	8	0.761	14	4.7E-01	0	0.359
3′ UTR stop codon density	0.067	0.070	0.052	0.066	0.976	0.066	0.859
Polycistronic genes	31	1	0.105	0	0.790	0	0.836
Up-regulated in *Upf2^25G^*	n/a	n/a	n/a	24	< 2e-16	n/a	n/a

NMD, nonsense-mediated mRNA decay; UTR, untranslated region; CDS, coding DNA sequence; n/a, not applicable.

a Genes that neither are up-regulated in *Upf2^25G^/Y* mutants nor undergo increased decay upon *Upf2* reactivation.

b Count or average for the indicated feature.

c Genes up-regulated in *Upf2^25G^/Y* mutants.

d *P* value based on logistic regression compared with nonresponding genes. Compared features are based on FlyBase annotations (R5.45); 3′ UTR length is the length of the longest annotated 3′ UTR in nucleotides; read-through candidates as defined in (Jungreis *et al.* 2011); free energy as calculated using CentroidFold (Hamada *et al.* 2009)

e Genes that undergo decay upon *Upf2* reactivation.

f Genes both up-regulated in *Upf2^25G^/Y* mutants and that undergo decay upon *Upf2* reactivation. Only genes that were detected in reactivation microarray experiments are tabulated.

### Comparison of *in vivo* NMD target analysis to cell-culture analysis

Previously, the most expansive analysis of NMD targeting in *Drosophila* was performed in S2 cell culture (Rehwinkel *et al.* 2005). This analysis identified a set of 184 “core” NMD targets, defined as the up-regulated genes after independent RNAi-mediated silencing of all six known NMD factors in *Drosophila*. Of these 184 genes, 68 passed our data quality control tests, thus allowing us to compare these genes to our NMD-regulated candidates. We find that these overlap between the data sets is relatively small, with the majority genes up-regulated in S2 cells not identified as up-regulated in our *Upf2^25G^* mutant, or identified as reactivation targets (Figure S3). This discordance between our NMD targeting data collected in whole animals *vs.* data from cell culture experiments is likely a reflection of the tissue, physiological, and developmental stage differences between these two experimental setups.

### NMD does not directly regulate genomic load *in vivo*

**Figure 4:**
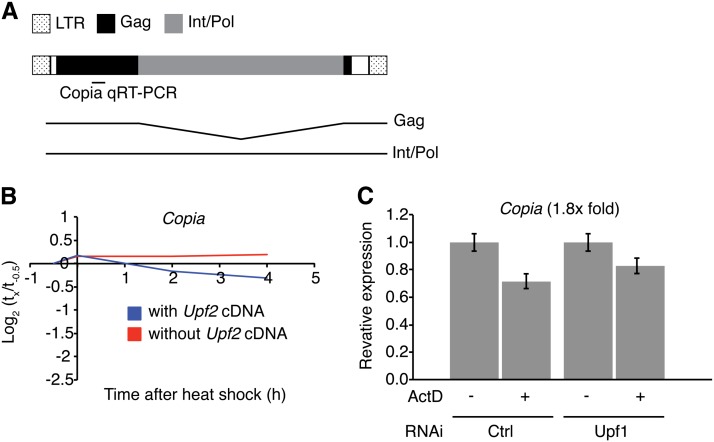
*Copia* RNA levels are indirectly regulated by the NMD pathway. (A) *Copia* genomic (boxes) and transcript structures. Also indicated is the quantitative reverse-transcription polymerase chain reaction (qRT-PCR) amplicon. (B) qRT-PCR analysis of *Copia* of the same time course described in Figure 2. (C) *Copia* levels in S2 cells, measured by qRT-PCR, using the assay described Figure S2 (fold change after *Upf1* depletion indicated in parentheses). Error bars represent ± 1 SD.

A proposed role of the NMD pathway is to rid the cell of aberrant transcripts, represented in part by transposable elements (TEs), and sporadic transcripts containing nonsense mutations that arise from errors in transcription or splicing (Mendell *et al.* 2004). A striking finding of our RNA-seq analysis of *Upf2* mutant larvae was a massive increase in reads mapping to the endogenous TE *Copia*. In control *Upf2^+^* larvae, sequences derived from this LTR-family transposon comprised 0.042% of all mappable reads. However, in *Upf2^25G^/Y*, *Copia* represented 0.77% of all reads (Table S1), a 17.4-fold increase. To test whether *Copia* RNA is a direct target of NMD-mediated degradation, we examined our reactivation samples. Because *Copia* is not represented on the microarrays, we used for global reactivation analysis, we used qRT-PCR to measure *Copia* mRNA in the reactivation time course (Figure 4). *Copia* undergoes an alternative splicing event to generate two major mRNA species (Brierley and Flavell 1990) and we used an qRT-PCR amplicon that detects both of these RNAs (Figure 4A). We found that *Copia* did not behave like a direct NMD pathway target in this assay, as *Copia* levels did not decrease significantly during our reactivation time course (Figure 4B). This conclusion was corroborated from results obtained in S2 cells, where RNAi-mediated depletion of *Upf1* increased *Copia* expression levels but did not alter the decay rate of *Copia* RNA (Figure 4C). Thus, we conclude that *Copia* transcript levels are regulated by processes downstream of direct NMD targeting, as we have found for the majority of genes that are expressed at increased levels in NMD mutant backgrounds.

To test whether *Upf2^25G^/Y* animals have an overall increase in mRNAs that contain PTCs, we analyzed the proportion of reads in our RNA-seq data that encode for nonsense transcripts (Table S3). We found that such reads comprise 0.048% of all reads mapping to mRNA in *Upf2^25G^*, compared to 0.046% in controls. Thus, we found no significant difference in the proportion of nonsense reads between *Upf2^25G^* and control, suggesting that such mutant transcripts do not accumulate in NMD mutants.

## Discussion

Although NMD has been established as an important pathway for posttranscriptional gene regulation in eukaryotes, the identities of the directly regulated genes as well as the mechanism of targeting have remained elusive. Identifying the direct targets has been particularly challenging in intact, multicellular organisms. In this work, we have used reactivation of the NMD pathway in an NMD-defective *Drosophila* mutant to identify directly targeted genes. Transcripts that rapidly decrease in abundance upon pathway reactivation are strong candidates for direct targets of NMD-mediated degradation. We have identified 168 candidate direct NMD targets, indicating that NMD regulates a significant proportion of the *Drosophila* genome. This number may, however, be an underestimate due to intrinsic limitations to our approach. For instance, we have examined NMD targeting at one specific stage of development, so we have not identified NMD targeting of genes that are either not transcribed at this stage, or transcribed but not translated, since NMD targeting is translation dependent. Thus, this group of 168 genes likely represents a lower limit to the number of genes directly targeted by the NMD pathway.

In addition to the reactivation assay, we have also measured the change in steady-state expression level of all transcripts in NMD-defective animals. A comparison of the reactivation and steady-state analysis leads to two significant conclusions. First, we found that only 16% of genes that are overexpressed at steady-state levels in the NMD mutant behave like direct targets in our reactivation assay. This finding suggests that, for most transcripts, the increased expression observed in the NMD mutant is due to indirect effects caused by NMD pathway disruption. Such a result was not unexpected, as it is likely that changes in direct target expression will lead to changes in downstream genes, either through specific regulation by overexpressed direct targets, or through changes in organismal development or physiology. In yeast, 45–54% of genes up-regulated in NMD mutants are direct targets (Guan *et al.* 2006; Johansson *et al.* 2007). The finding that in whole *Drosophila*, loss of NMD shows a greater percentage of up-regulated indirect targets may be due to the diversity of tissue-specific physiologic response brought about by loss of NMD (Weischenfeldt *et al.* 2008; Bruno *et al.* 2011; Colak *et al.* 2013). This scenario is important to consider when developing systemically administered therapies designed to modulate NMD activity (Durand *et al.* 2007).

Our second conclusion is more surprising. We found that 81% of candidate direct targets do not show altered steady-state expression in our NMD mutant. This implies there exist feedback mechanisms that act to renormalize the expression of many NMD-target genes when NMD function is compromised. Many gene expression networks contain negative regulatory loops that act to restore expression to physiological levels upon perturbation of pathway components (Becskei and Serrano 2000). This feedback could function indirectly, for instance at the transcriptional level, but in the case of NMD, the feedback could also be working at the level of NMD pathway activity. The NMD effectors *Smg5* and *Smg6* are direct NMD targets, both in mammalian and fly cells (Rehwinkel *et al.* 2005; Huang *et al.* 2011; Yepiskoposyan *et al.* 2011), and our reactivation experiments demonstrate direct targeting of these genes in intact *Drosophila*. The up-regulation of *Smg5* and/or *Smg6* may compensate for the partial loss of *Upf2* function in our *Upf2* hypomorph, restoring gene expression of many direct targets to homeostatic levels. However, upon reactivation of *Upf2*, these direct targets are poised for rapid degradation due to increased expression of *Smg5*, *Smg6*, and other potential NMD factors. Such a model may account for why in yeast a greater percentage of direct NMD target genes show increased expression in NMD mutants (Johansson *et al.* 2007), since there is no evidence for NMD pathway autoregulation in this organism. That some directly targeted genes do show up-regulation in *Upf2^25G^* may be indicative of a greater dependence of these genes on core NMD factors for their degradation, as opposed to auxiliary regulators, as has been observed in a number of studies (Chen *et al.* 2005; Metzstein and Krasnow 2006; Avery *et al.* 2011; Huang *et al.* 2011; Frizzell *et al.* 2012; Metze *et al.* 2013). A final speculative possibility is that NMD factors may also be required for transcription of some direct NMD-mediated mRNA decay substrates, as has been shown for yeast proteins involved in general 5′-3′ RNA degradation (Haimovich *et al.* 2013; Sun *et al.* 2013). In this scenario, loss of NMD factors results in reduced transcription of these NMD targets, compensating for the concurrent increase in mRNA stability, which results in unchanged steady-state expression levels. A number of NMD factors are known to shuttle between the cytoplasm and the nucleus (Isken and Maquat 2008), and some are known to have nuclear functions (Brogna 1999; de Turris *et al.* 2011; Varsally and Brogna 2012).

Although our conclusions that most genes up-regulated in NMD-defective *Drosophila* are not direct targets of the NMD pathway, and that many direct targets are not up-regulated, differs what has been found in yeast (Guan *et al.* 2006; Johansson *et al.* 2007), they are similar to findings in mammalian cell culture (Tani *et al.* 2012a). Tani *et al.* (2012a) found that 23% of genes in HeLa cells that are up-regulated under conditions of NMD deficiency appear to be direct NMD targets, similar to the 16% we have shown here. They also observed that the great majority of genes (90%) that displayed NMD pathway-dependent decay, and thus appeared to be direct degradation targets, had no change in steady-state expression levels when the NMD pathway was inactivated. Again, this is very similar to what we have found in intact *Drosophila*, in which 81% of putative direct targets have no significant change in expression. That such similar results were found using different methodologies in diverse organisms suggests that the phenomenon of feedback regulation normalizing levels of direct NMD targets is a general property of the NMD pathway in metazoans.

Although a rapid decrease in mRNA expression levels upon Upf2 reactivation suggests that the gene is a direct NMD target, it remains possible that the expression levels of some of our identified genes decrease due to a non NMD mechanism. This alternative mechanism is likely to depend on activation of a secondary RNA decay pathway, as transcriptional changes in response to Upf2 expression is unlikely in itself to lead to the rapid transcript decay we observe. Future experiments, such as detection of specific NMD-dependent decay intermediates, could be used to address this alternative possibility; such an approach has been well established in cell culture, but not previously in intact animals.

Most studies seeking to globally identify NMD targets have used overexpression as the main criteria for being an NMD target (Lelivelt and Culbertson 1999; He *et al.* 2003; Mendell *et al.* 2004; Rehwinkel *et al.* 2005; Guan *et al.* 2006; Metzstein and Krasnow 2006; Wittmann *et al.* 2006; Barberan-Soler *et al.* 2009; Ramani *et al.* 2009; McIlwain *et al.* 2010; Nicholson *et al.* 2010; Yepiskoposyan *et al.* 2011; Hurt *et al.* 2013; Matia-González *et al.* 2013). Cataloging NMD targets in this way produces a list with a mix of indirect and direct NMD targets. This in turn, convolutes the correlation between 3′ UTR length and the likelihood of being an *in vivo* NMD target, as has been observed in previous analyses (Rehwinkel *et al.* 2005; Hansen *et al.* 2009; Ramani *et al.* 2009; Yepiskoposyan *et al.* 2011; Rayson *et al.* 2012). In experimental situations, long 3′ UTRs generally make transcripts sensitive to NMD (Kertész *et al.* 2006; Behm-Ansmant *et al.* 2007a; Longman *et al.* 2007; Singh *et al.* 2008; Eberle *et al.* 2009; Hogg and Goff 2010). Our examination of natural *in vivo* NMD targets supports this conclusion: we find that candidate direct NMD targets have considerably longer 3′ UTRs on average than nondirect targets. Almost 50% of candidate direct target transcripts have a 3′ UTR over 420 bases in length, a value suggested to be a threshold between NMD resistance and sensitivity (Singh *et al.* 2008), compared with only 31% of nontargeted transcripts. However, as has been found in mammalian cells, we find that a large number of transcripts that have long 3′ UTRs are not subject to NMD (with the caveat that, in most cases, defining a transcript as NMD sensitive in mammalian cells is based on increased expression when the NMD pathway is disabled). Singh *et al.* (2008) have proposed such long but NMD-insensitive transcripts contain specific NMD-protective sequences. Conversely, we also find that there are many genes with short 3′ UTRs that appear to be direct NMD targets. Since most models invoke a long 3′ UTR as the trigger for NMD targeting, how structurally normal, short 3′ UTRs could be targeted is unclear. We propose these transcripts may contain specific sequence features that stimulate NMD targeting, analogous to yeast downstream elements (Peltz *et al.* 1993; González *et al.* 2001). Although such elements are not required to trigger decay in NMD-sensitive transcripts with long 3′ UTRs, they could be key to targeting mRNAs with short 3′ UTRs. Furthermore, the decay rates of both mutant PTC-containing and endogenous transcripts are often quite variable when measured experimentally, a phenomenon that could be explained by the presence or absence of DSE-like elements in these transcripts.

Finally, as described previously, our results regarding the number of direct targets of the NMD pathway correlate well with the findings in HeLa cells. However, the results from this study differed from ours in one significant aspect: in HeLa cells direct targets were found to have shorter 3′ UTR lengths than nontargets (Tani *et al.* 2012a), a finding contradictory to our own and unexpected given current models of NMD targeting. A possible explanation for this discrepancy is that cell transformation is associated with a global alteration in 3′ UTR length (Mayr and Bartel 2009). Potentially, the 3′ UTR lengths of NMD targeted transcripts in HeLa cells systematically differ from the annotated human genome reference set, explaining the discordance. It will be important in the future to examine NMD targeting in nontransformed mammalian cells to determine the contribution of 3′ UTR length to NMD sensitivity.

One model for why the NMD pathway is required for viability is that NMD functions to suppress transcriptome noise, such as the noise arising from endogenous TEs or erroneously generated PTC-bearing mRNAs. However, although we find that the TE *Copia* is highly up-regulated in *Upf2^25G^* mutants, Copia RNA is not a direct NMD target *in vivo*. Therefore, *Copia* up-regulation is likely due to processes downstream of NMD, such as stress responses, which are known to up-regulate *Copia* in other experimental situations (Strand and McDonald 1985). Moreover, we find no significant enrichment in reads that map to PTC-generating transcripts in *Upf2^25G^*, suggesting that NMD does not function to suppress such transcripts *in vivo*. Since we find no evidence that NMD suppresses genomic noise *in vivo*, we favor a model in which NMD is required for viability through regulation of particular critical NMD target genes. Therefore, the understanding of the *in vivo* roles of the NMD pathway will require the identification of these targets, particularly those that display increased expression in NMD mutants, as it is likely that this increased expression mediates the biological defects observed in NMD-defective organisms and cells. Our analysis indicates that this is a relatively short list; we find only 24 genes appear to be both be overexpressed and be direct targets at the developmental stage we examined. The continued analysis of these 24 genes should reveal important insights into NMD-dependent roles in normal development and physiology.

## Supplementary Material

Supporting Information
