# Supplementary material for: In Vivo Determination of Direct Targets of the Nonsense-Mediated Decay Pathway in Drosophila
Source: G3 (Bethesda). 2014 Jan 15;4(3):485–96. doi: 10.1534/g3.113.009357 (PMC3962487; doi:10.1534/g3.113.009357)
Supplement: Supporting Information [file supp_4_3_485__index.html]

In Vivo Determination of Direct Targets of the Nonsense-Mediated Decay Pathway in Drosophila — Supporting Information 

# *In Vivo* Determination of Direct Targets of the Nonsense-Mediated Decay Pathway in *Drosophila*

## Supporting Information for Chapin *et al.*, 2014

**Files in this Data Supplement:**

- Supporting Information - Figures S1-S3, Files S1-S5, and Tables S1-S4 (PDF, 875 KB)
- Figure S1 - Heat shock activation of *Upf2*. (PDF, 85 KB)
- Figure S2 - Stabilization of direct NMD targets in cultured S2 cells following RNAi-mediated knockdown of *Upf1*. (PDF, 90 KB)
- Figure S3 - Comparison of NMD target genes from S2 cells and intact *Drosophila*. (PDF, 465 KB)
- Table S1 - Sequencing reads for all four replicates. (PDF, 62 KB)
- Table S2 - Significantly upregulated and downregulated genes at p <0.01. (PDF, 66 KB)
- Table S3 - Reads mapping to PTC-harboring transcripts in *Upf225G* and control. (PDF, 69 KB)
- Table S4 - Sequences of primers used in this study. (PDF, 312 KB)
- File S1 - List of all genes analyzed (.xlsx, 1 MB)
- File S2 - List of significantly up and down regulated genes (.xlsx, 109 KB)
- File S3 - All genes analyzed in reactivation experiments (.xlsx, 964 KB)
- File S4 - All identified reactivation targets (.xlsx, 76 KB)
- File S5 - All genes analyzed for features analysis (.xlsx, 768 KB)
