# Supplementary material for: In Vivo Determination of Direct Targets of the Nonsense-Mediated Decay Pathway in Drosophila
Source: G3 (Bethesda). 2014 Jan 15;4(3):485–96. doi: 10.1534/g3.113.009357 (PMC3962487; doi:10.1534/g3.113.009357)
Supplement: Supporting Information [file supp_g3.113.009357_009357SI.pdf]

G3: Genes | Genomes | Genetics

In vivo determination of direct targets of the nonsense mediated decay pathway in *Drosophila*

Alex Chapin[*,**], Hao Hu[*,1,**], Shawn G. Rynearson[*], Julie Hollien[§], Mark Yandell[*] and Mark M. Metzstein[*]

[*]Department of Human Genetics, [§]Department of Biology, University of Utah, Salt Lake City, UT 84112, USA
[1]Current address: Department of Epidemiology, The University of Texas MD Anderson Cancer Center, Houston, Texas 77030, USA
[**]These authors contributed equally to this work

Author for correspondence:
Mark M. Metzstein
15 N 2030 E
Salt Lake City, UT 84112
USA
Email: markm@genetics.utah.edu
Phone: (801)-585-9941
FAX: (801)-585-3214

Short read archive:
SRR896609
SRR896616
SRR503415
SRR503416

GEO:
GSE47979

DOI: 10.1534/g3.113.009357

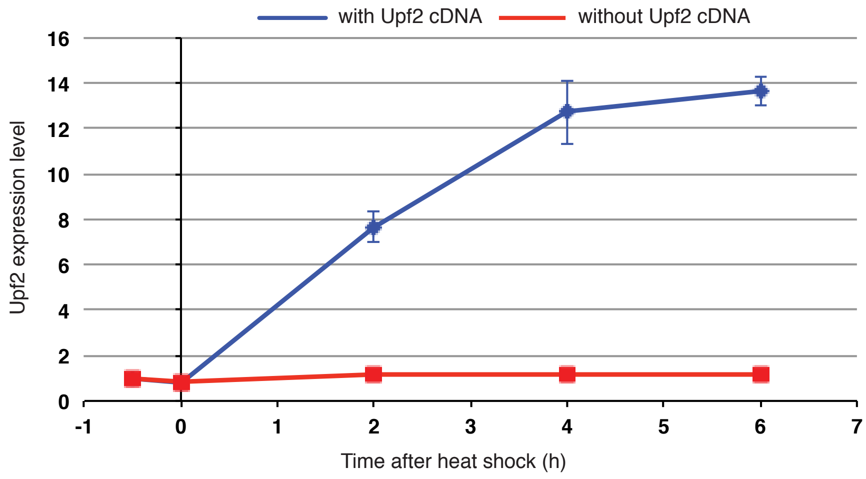

Figure S1 Heat shock activation of *Upf2*. Fold change in expression of *Upf2* mRNA relative to pre-heat shock levels as measured by qRT-PCR. Genotypes are *Upf2^{25G}/Y ; UAS:Upf2/ hsp70:GAL4* (experimental, blue) or *Upf2^{25G}/Y ; +/ hsp70:GAL4* (control, red). Error bars represent ± 1 SD.

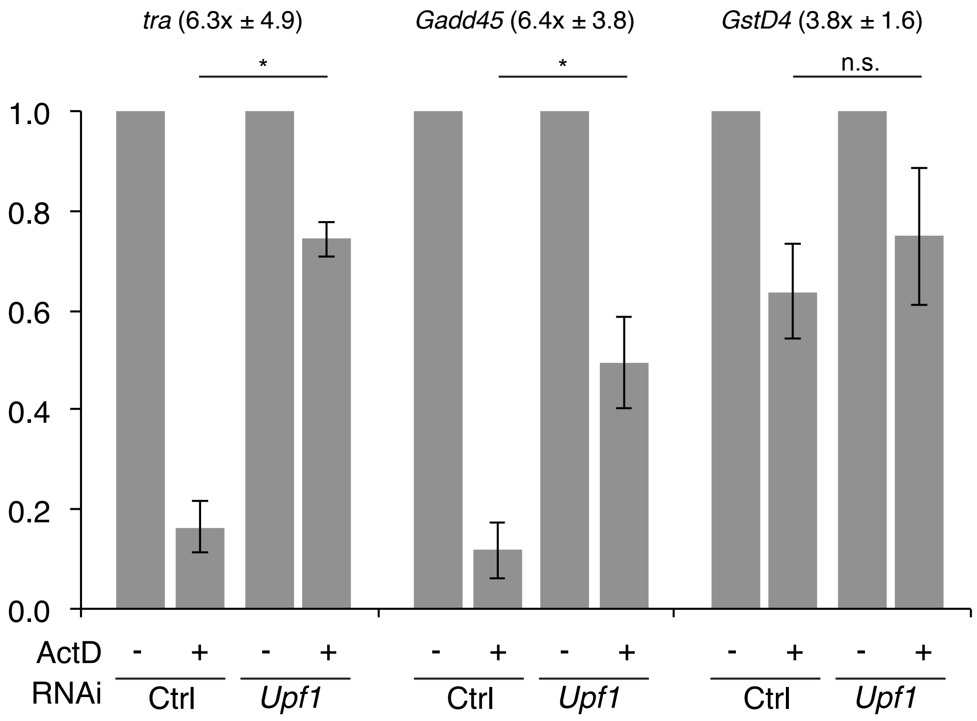

Figure S2 Stabilization of direct NMD targets in cultured S2 cells following RNAi-mediated knockdown of *Upf1*. Cells were mock-treated (Ctrl) or depleted of *Upf1* using RNAi, then incubated in the presence or absence of ActD for one hour. Expression levels of the indicated genes were measured by qRT-PCR; shown are the averages for two independent experiments. Expression levels of *tra, Gadd45* and *GstD4* are all increased by *Upf1* depletion (fold change indicated in parentheses). While the stability of the direct NMD targets *Gadd45* and *tra* is increased by this treatment, the stability of the indirect NMD target *GstD4* is not. Error bars represent ± 1 SD. * indicates $p < 0.05$; n.s., $p = 0.46$ (Student's T-test).

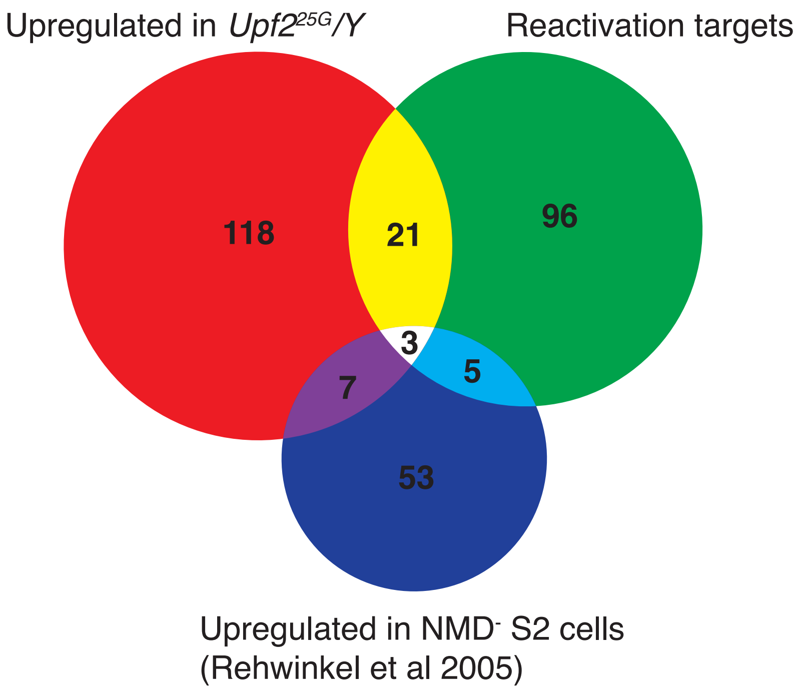

Figure S3 Comparison of NMD target genes from S2 cells and intact *Drosophila*. Overlap of genes upregulated in *Upf2*^{25G}, reactivation targets, and a core set of upregulated NMD target genes as defined by Rehwinkel *et. al.,* (2005). The three genes identified in all experiments (white, central area) are *Smg5, Smg6,* and *Gadd45.*

Files S1-S5

Available for download as Excel files at http://www.g3journal.org/lookup/suppl/doi:10.1534/g3.113.009357/-/DC1

File S1 List of all genes analyzed

File S2 List of significantly up and down regulated genes

File S3 All genes analyzed in reactivation experiments

File S4 All identified reactivation targets

File S5 All genes analyzed for features analysis

Table S1 Sequencing reads for all four replicates. All read numbers are reported in millions with the exception of *Copia*.
† Total sequenced reads. § reads mapped to reference exome. ‡ Reads mapped to mRNA (i.e. excluding rRNAs and tRNAs).

Genotype	Replicate	Total reads†	Mapped reads§	Mapped/total	mRNA‡	mRNA/ mapped	Total copia reads	% of copia/ mapped reads
y w FRT^{19A}/Y	A	136.2	114.7	84.21%	7.5	6.54%	45832	0.040%
	B	139.7	123.9	88.69%	10.8	8.71%	53576	0.043%
Upf2^{25G}/Y	A	134.2	104.6	77.97%	10.0	9.54%	590497	0.565%
	B	102.8	43.4	42.23%	3.9	8.88%	544427	1.254%

Table S2 Significantly upregulated and downregulated genes at p <0.01. Expression is given as the number of reads per million mapped read per kilobase exon model (FPKM). Top 10 of each class are shown in each table. Full lists are available in File S2.

Top upregulated gene p <0.01						
FBgn	Gene name	FPKM control	FPKM *Upf2*^{25G}	Fold change (log_2)	p value	3' UTR length
FBgn0028396	TotA	0.14	45.39	8.37	3.81E-66	153
FBgn0010042	GstD6	0.67	38.57	5.85	2.46E-49	88
FBgn0010041	GstD5	3.55	142.07	5.32	4.26E-50	na
FBgn0041183	TepI	0.03	1.02	5.16	1.55E-23	63
FBgn0034480	CG16898	4.62	161.02	5.12	5.29E-49	72
FBgn0039316	CG11893	6.95	210.94	4.92	1.70E-46	126
FBgn0040104	lectin-24A	1.48	44.52	4.91	6.76E-42	80
FBgn0037850	CG14695	0.39	9.29	4.58	1.56E-28	200
FBgn0010039	GstD3	2.60	53.19	4.36	5.57E-35	84
FBgn0052437	CG32437	0.07	1.42	4.27	3.35E-16	51

Top downregualted genes p <0.01						
FBgn	Gene name	FPKM control	FPKM *Upf2*^{25G}	Fold change (Log_2)	p value	3' UTR length
FBgn0023495	*Lip3*	7.96	0.27	-4.88	3.62E-30	91
FBgn0033726	*Cpr49Ad*	4.75	0.20	-4.57	7.26E-15	na
FBgn0039476	*CG6271*	31.50	1.44	-4.45	6.32E-35	25
FBgn0036622	*CG4753*	2.77	0.13	-4.40	1.05E-07	153
FBgn0261997	*CG42815*	16.44	0.80	-4.37	1.24E-20	na
FBgn0035790	*Cyp316a1*	0.20	0.01	-4.35	4.37E-03	na
FBgn0031741	*CG11034*	0.36	0.02	-4.28	6.86E-07	na
FBgn0013772	*Cyp6a8*	3.27	0.18	-4.21	2.70E-21	230
FBgn0038095	*Cyp304a1*	2.02	0.12	-4.12	8.45E-17	158
FBgn0262146	*MtnE*	97.04	6.70	-3.86	6.24E-29	150

Table S3 Reads mapping to PTC-harboring transcripts in *Upf2*^{25G} and control. †Reads which contain sequence variants that, when mapped, generate a PTC-bearing isoform. *All reads which map to mRNA. Each replicates for each genotype is shown.

	†PTC reads	*Total mRNA reads	Proportion PTC reads
FRT^{19A}/*Y*_A	3639	7505322	0.0485%
FRT^{19A}/*Y*_B	4597	10793541	0.0426%
Upf2^{25G}/*Y*_A	4811	9975004	0.0482%
Upf2^{25G}/*Y*_B	1840	3852102	0.0478%

Table S4 Sequences of primers used in this study

Name	Sequence
Upf2xF1	GAGCGGCCGCATGCTAGCCAACGATTCTG
Upf2xR1	GGCATTTTTACGTACTAAGTAGGCCGGTATCGATGTCGTTGTC
Upf2xF2	GACAACGACATCGATACCGGCCTACTTAGTACGTAAAAATGCC
Upf2xR2	CTCCCGGGCGTGTATCTTATTTATTCATC
qGadd45_F1	CATCAACGTGCTCTCCAAGTC
qGadd45_R1	CGTAGATGTCGT TCTCGTAGC
qRP49_F	ATGCTAAGCTGTCGCACAAA
qRP49_R	CGATGTTGGGCATCAGATAC
qCopia_F1	GGCGTTTGTGAAAAATAGATTGC
qCopia_R1	GATCGCGTTCATAACTTTCTTGC
Upf1_RNAi_F	TTAATACGACTCACTATAGGGAGA TCGGTGGATCTTCTCAGTTAGC
Upf1_RNAi_R	TTAATACGACTCACTATAGGGAGA ACCAAAAACTGAAGGAGTCTGC
qtra_F	GTAGCCAAATCGCGGAACTC
qtra_R	ATACCAAAGGCTACCACGTCCTC
