# Supplementary material for: In Vivo Determination of Direct Targets of the Nonsense-Mediated Decay Pathway in Drosophila
Source: G3 (Bethesda). 2014 Jan 15;4(3):485–96. doi: 10.1534/g3.113.009357 (PMC3962487; doi:10.1534/g3.113.009357)
Supplement: Supporting Information [file supp_g3.113.009357_FigureS1.pdf]

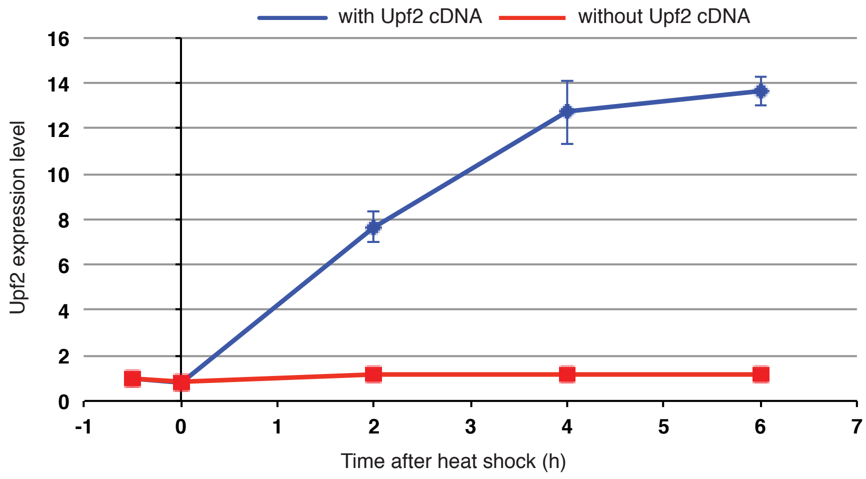

Figure S1 Heat shock activation of *Upf2*. Fold change in expression of *Upf2* mRNA relative to pre-heat shock levels as measured by qRT-PCR. Genotypes are *Upf2*^{25G}*/Y* ; *UAS:Upf2/ hsp70:GAL4* (experimental, blue) or *Upf2*^{25G}*/Y* ; *+/ hsp70:GAL4* (control, red). Error bars represent ± 1 SD.
