# Supplementary material for: In Vivo Determination of Direct Targets of the Nonsense-Mediated Decay Pathway in Drosophila
Source: G3 (Bethesda). 2014 Jan 15;4(3):485–96. doi: 10.1534/g3.113.009357 (PMC3962487; doi:10.1534/g3.113.009357)
Supplement: Supporting Information [file supp_g3.113.009357_FigureS2.pdf]

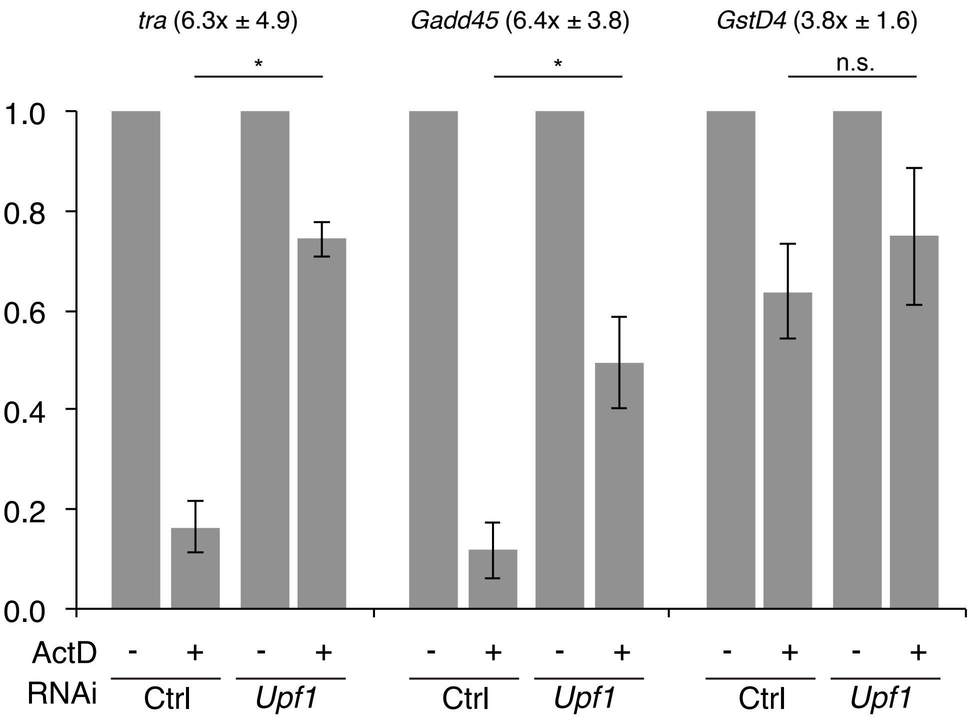

Figure S2 Stabilization of direct NMD targets in cultured S2 cells following RNAi-mediated knockdown of *Upf1*. Cells were mock-treated (Ctrl) or depleted of *Upf1* using RNAi, then incubated in the presence or absence of ActD for one hour. Expression levels of the indicated genes were measured by qRT-PCR; shown are the averages for two independent experiments. Expression levels of *tra, Gadd45* and *GstD4* are all increased by *Upf1* depletion (fold change indicated in parentheses). While the stability of the direct NMD targets *Gadd45* and *tra* is increased by this treatment, the stability of the indirect NMD target *GstD4* is not. Error bars represent ± 1 SD. * indicates $p < 0.05$; n.s., $p = 0.46$ (Student's T-test).
