# Supplementary material for: In Vivo Determination of Direct Targets of the Nonsense-Mediated Decay Pathway in Drosophila
Source: G3 (Bethesda). 2014 Jan 15;4(3):485–96. doi: 10.1534/g3.113.009357 (PMC3962487; doi:10.1534/g3.113.009357)
Supplement: Supporting Information [file supp_g3.113.009357_FigureS3.pdf]

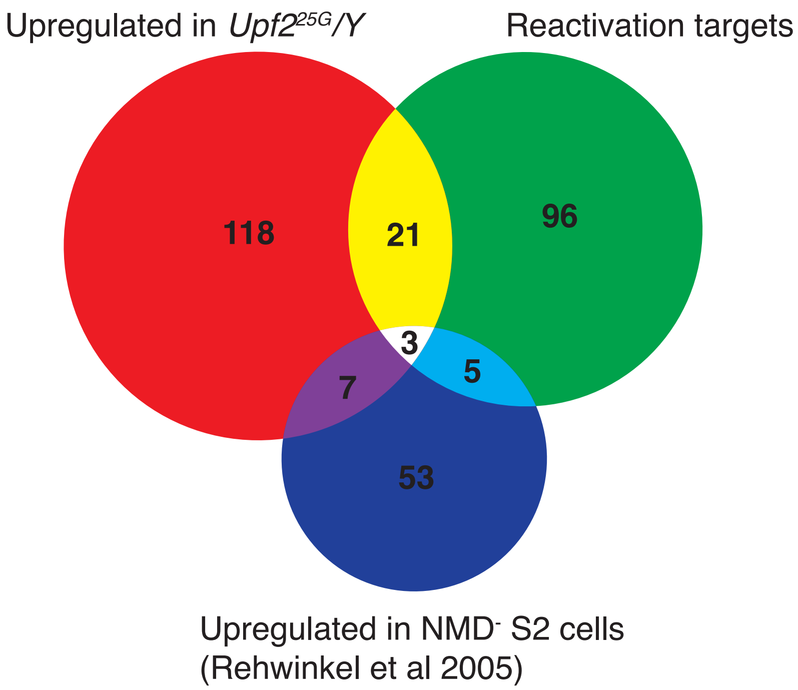

Figure S3 Comparison of NMD target genes from S2 cells and intact *Drosophila*. Overlap of genes upregulated in *Upf2*^{25G}, reactivation targets, and a core set of upregulated NMD target genes as defined by Rehwinkel *et. al.,* (2005). The three genes identified in all experiments (white, central area) are *Smg5, Smg6,* and *Gadd45.*
