# Supplementary material for: In Vivo Determination of Direct Targets of the Nonsense-Mediated Decay Pathway in Drosophila
Source: G3 (Bethesda). 2014 Jan 15;4(3):485–96. doi: 10.1534/g3.113.009357 (PMC3962487; doi:10.1534/g3.113.009357)
Supplement: Supporting Information [file supp_g3.113.009357_TableS1.pdf]

Table S1 Sequencing reads for all four replicates. All read numbers are reported in millions with the exception of *Copia*.
† Total sequenced reads. § reads mapped to reference exome. ‡ Reads mapped to mRNA (i.e. excluding rRNAs and tRNAs).

Genotype	Replicate	Total reads†	Mapped reads§	Mapped/total	mRNA‡	mRNA/ mapped	Total copia reads	% of copia/ mapped reads
y w FRT^{19A}/Y	A	136.2	114.7	84.21%	7.5	6.54%	45832	0.040%
	B	139.7	123.9	88.69%	10.8	8.71%	53576	0.043%
Upf2^{25G}/Y	A	134.2	104.6	77.97%	10.0	9.54%	590497	0.565%
	B	102.8	43.4	42.23%	3.9	8.88%	544427	1.254%
