# Supplementary material for: In Vivo Determination of Direct Targets of the Nonsense-Mediated Decay Pathway in Drosophila
Source: G3 (Bethesda). 2014 Jan 15;4(3):485–96. doi: 10.1534/g3.113.009357 (PMC3962487; doi:10.1534/g3.113.009357)
Supplement: Supporting Information [file supp_g3.113.009357_TableS2.pdf]

Table S2 Significantly upregulated and downregulated genes at p <0.01. Expression is given as the number of reads per million mapped read per kilobase exon model (FPKM). Top 10 of each class are shown in each table. Full lists are available in File S2.

Top upregulated gene p <0.01						
FBgn	Gene name	FPKM control	FPKM $Upf2^{25G}$	Fold change (log_2)	p value	3' UTR length
FBgn0028396	TotA	0.14	45.39	8.37	3.81E-66	153
FBgn0010042	GstD6	0.67	38.57	5.85	2.46E-49	88
FBgn0010041	GstD5	3.55	142.07	5.32	4.26E-50	na
FBgn0041183	TepI	0.03	1.02	5.16	1.55E-23	63
FBgn0034480	CG16898	4.62	161.02	5.12	5.29E-49	72
FBgn0039316	CG11893	6.95	210.94	4.92	1.70E-46	126
FBgn0040104	lectin-24A	1.48	44.52	4.91	6.76E-42	80
FBgn0037850	CG14695	0.39	9.29	4.58	1.56E-28	200
FBgn0010039	GstD3	2.60	53.19	4.36	5.57E-35	84
FBgn0052437	CG32437	0.07	1.42	4.27	3.35E-16	51

Top downregualted genes p <0.01						
FBgn	Gene name	FPKM control	FPKM $Upf2^{25G}$	Fold change (Log_2)	p value	3' UTR length
FBgn0023495	*Lip3*	7.96	0.27	-4.88	3.62E-30	91
FBgn0033726	*Cpr49Ad*	4.75	0.20	-4.57	7.26E-15	na
FBgn0039476	*CG6271*	31.50	1.44	-4.45	6.32E-35	25
FBgn0036622	*CG4753*	2.77	0.13	-4.40	1.05E-07	153
FBgn0261997	*CG42815*	16.44	0.80	-4.37	1.24E-20	na
FBgn0035790	*Cyp316a1*	0.20	0.01	-4.35	4.37E-03	na
FBgn0031741	*CG11034*	0.36	0.02	-4.28	6.86E-07	na
FBgn0013772	*Cyp6a8*	3.27	0.18	-4.21	2.70E-21	230
FBgn0038095	*Cyp304a1*	2.02	0.12	-4.12	8.45E-17	158
FBgn0262146	*MtnE*	97.04	6.70	-3.86	6.24E-29	150
