# Supplementary material for: In Vivo Determination of Direct Targets of the Nonsense-Mediated Decay Pathway in Drosophila
Source: G3 (Bethesda). 2014 Jan 15;4(3):485–96. doi: 10.1534/g3.113.009357 (PMC3962487; doi:10.1534/g3.113.009357)
Supplement: Supporting Information [file supp_g3.113.009357_TableS3.pdf]

Table S3 Reads mapping to PTC-harboring transcripts in *Upf2*^{25G} and control. †Reads which contain sequence variants that, when mapped, generate a PTC-bearing isoform. *All reads which map to mRNA. Each replicates for each genotype is shown.

	†PTC reads	*Total mRNA reads	Proportion PTC reads
FRT^{19A}/*Y*_A	3639	7505322	0.0485%
FRT^{19A}/*Y*_B	4597	10793541	0.0426%
Upf2^{25G}/*Y*_A	4811	9975004	0.0482%
Upf2^{25G}/*Y*_B	1840	3852102	0.0478%
