# Supplementary material for: In Vivo Determination of Direct Targets of the Nonsense-Mediated Decay Pathway in Drosophila
Source: G3 (Bethesda). 2014 Jan 15;4(3):485–96. doi: 10.1534/g3.113.009357 (PMC3962487; doi:10.1534/g3.113.009357)
Supplement: Supporting Information [file supp_g3.113.009357_TableS4.pdf]

Table S4 Sequences of primers used in this study

Name	Sequence
Upf2xF1	GAGCGGCCGCATGCTAGCCAACGATTCTG
Upf2xR1	GGCATTTTTACGTACTAAGTAGGCCGGTATCGATGTCGTTGTC
Upf2xF2	GACAACGACATCGATACCGGCCTACTTAGTACGTAAAAATGCC
Upf2xR2	CTCCCGGGCGTGTATCTTATTTATTCATC
qGadd45_F1	CATCAACGTGCTCTCCAAGTC
qGadd45_R1	CGTAGATGTCGT TCTCGTAGC
qRP49_F	ATGCTAAGCTGTCGCACAAA
qRP49_R	CGATGTTGGGCATCAGATAC
qCopia_F1	GGCGTTTGTGAAAAATAGATTGC
qCopia_R1	GATCGCGTTCATAACTTTCTTGC
Upf1_RNAi_F	TTAATACGACTCACTATAGGGAGA TCGGTGGATCTTCTCAGTTAGC
Upf1_RNAi_R	TTAATACGACTCACTATAGGGAGA ACCAAAAACTGAAGGAGTCTGC
qtra_F	GTAGCCAAATCGCGGAACTC
qtra_R	ATACCAAAGGCTACCACGTCCTC
